# Ion Channel Involvement in Tumor Drug Resistance

**DOI:** 10.3390/jpm12020210

**Published:** 2022-02-03

**Authors:** Concetta Altamura, Paola Gavazzo, Michael Pusch, Jean-François Desaphy

**Affiliations:** 1 Section of Pharmacology, Department of Biomedical Sciences and Human Oncology, School of Medicine, University of Bari Aldo Moro, 70124 Bari, Italy; concetta.altamura@uniba.it; 2 Institute of Biophysics, National Research Council, 16149 Genoa, Italy; paola.gavazzo@ibf.cnr.it (P.G.); michael.pusch@ibf.cnr.it (M.P.)

**Keywords:** ion channel, cancer, drug resistance, chemotherapeutics

## Abstract

Over 90% of deaths in cancer patients are attributed to tumor drug resistance. Resistance to therapeutic agents can be due to an innate property of cancer cells or can be acquired during chemotherapy. In recent years, it has become increasingly clear that regulation of membrane ion channels is an important mechanism in the development of chemoresistance. Here, we review the contribution of ion channels in drug resistance of various types of cancers, evaluating their potential in clinical management. Several molecular mechanisms have been proposed, including evasion of apoptosis, cell cycle arrest, decreased drug accumulation in cancer cells, and activation of alternative escape pathways such as autophagy. Each of these mechanisms leads to a reduction of the therapeutic efficacy of administered drugs, causing more difficulty in cancer treatment. Thus, targeting ion channels might represent a good option for adjuvant therapies in order to counteract chemoresistance development.

## 1. Introduction

Drug resistance is the major limitation to success in antineoplastic therapies [1]. Intrinsic drug resistance, being present before the beginning of therapy, could be theoretically easily overcome by choosing the most adequate treatment, if available. However, acquired drug resistance, which develops along with therapy, represents a major issue since tumors can adapt to any treatment and turn back to progression after a period of stabilization or regression. Hence, although many tumors may respond drastically to initial treatment, recurrent disease will occur in many cases. It is estimated that over 90% of death in cancer patients is attributed to tumor drug resistance [2]. A plethora of cellular mechanisms can cause drug resistance, including modification of drug pharmacokinetics (metabolism and excretion), alteration of drug-receptor interaction, and activation of compensatory pathways.

Ion channels are transmembrane proteins allowing the selective flux of ions across membranes. Besides, their canonical role in regulating cell excitability in excitable tissues and fluid secretion in epithelia, there is now plenty of evidence that ion channels are also involved in modulating cell division, differentiation, migration, and death. As such, their involvement in tumor cell fate has been demonstrated in many cancer types [3,4,5,6,7,8]. Thus, many ongoing research projects are focusing on the evaluation of ion channels as biomarkers and druggable targets in cancer [9,10,11,12,13,14,15].

Recently, ion channels have also emerged as possible key actors in the mechanisms of drug resistance. Such effects may depend on the tumor type and on the tumor stage. These observations may pave the way for the development of personalized therapies aimed at impeding or, at least, slowing drug resistance development through modulation of ion channels. Ion channels are theoretically appealing drug targets since they can be easily modulated by small molecular-weight molecules when expressed on plasma membrane. Considering the arsenal of ion channel-targeting drugs already on the market for many diseases, there is the possibility of rapid translation to the oncologic patient through drug repurposing. If no drug is available yet, drug discovery should be promoted to broaden therapeutic options in refractory cancer. For instance, the search of monoclonal antibodies directed against ion channels is in the early stages of development. Here, we review current knowledge about plasma membrane ion channel involvement in the mechanisms of antineoplastic drug resistance. The review is divided in chapters for each ion channel family. Note that although some ligand-gated ion channels, such as nicotinic and purinergic P2X7 receptors, may play a role in cancer, these channels are not treated in this review [16,17].

## 2. Potassium Channels

With 78 human genes encoding the principal α-subunits, many auxiliary subunits, and a plethora of splice variants, potassium channels represent the largest and most diverse family among ion channels. Four classes of K^+^ channels have been defined: voltage-gated potassium channels (Kv), calcium-activated potassium channels (KCa), inward-rectifier potassium channels (Kir), and two-pore-domain potassium channels [18]. All of these channels play a critical role in the control of cell excitability as well as in cell volume regulation, proliferation, migration and apoptosis. Thus, altered expression of K^+^ channel has been widely associated with a growing number of diseases, including cancer [12,14,19,20,21,22,23,24].

Over the years, various groups tried to detail the cellular mechanisms linking K^+^ channels to chemotherapeutic resistance, demonstrating that both overexpression and reduction of K^+^ channels expression may be involved in chemosensitivity. Since the effects and the underlying mechanisms of potassium ion channels may vary between tumors, the main findings are summarized in Table 1.

### 2.1. Voltage-Gated Potassium Channels

The voltage-gated channel Kv11.1 (hERG1) represents one of the most studied K^+^ channels in cancer, being implicated in cell proliferation, apoptosis, angiogenesis, and metastasis of a number of cancers, including head and neck, gastric and colorectal cancers [36,37,38]. Generally, expression of Kv11.1 is selectively increased in cancer cells and the channel might be considered as a biomarker of cancer progression or a target for therapeutic intervention (for review see [39,40]).

A role for Kv11.1 in cancer cell response to drugs has been also suggested by a number of studies. For instance, the lung cancer A549 cell line that expresses low levels of hERG channels was less sensitive to vincristine, hydroxy-camptothecin and paclitaxel than the colorectal cancer HT-29 cell line expressing high levels of the channel [25]. In vitro and in vivo studies demonstrated that the treatment with cisplatin upregulated the expression of Kv11.1, both in gastric cancer cell line and in a rat model of gastric cancer [26]. Silencing of Kv11.1 expression with siRNA significantly reduced the chemosensitivity of gastric cancer cells to cisplatin by interfering with apoptosis mediated by members of the Bcl-2 family [26]. While these studies suggested that hERG channel inhibition might decrease cancer cell chemosensitivity, other studies supported the opposite conclusion. In acute lymphoblastic leukemia, leukemic cells were protected by bone marrow mesenchymal cells (MSC) thanks to their plasma membrane expression of a complex formed by β1-integrin, CXCR4, and the Kv11.1 channel. The use of different Kv11.1 inhibitors abrogated the protective effect of MSC and enhanced the cytotoxicity of doxorubicin, prednisone, or methotrexate, commonly used to treat leukemia [27]. In colorectal cancer, the use of Kv11.1 channel inhibitors on cisplatin-resistant colorectal cells promoted cisplatin uptake and enhanced apoptosis both in vitro and in a mouse model [28]. While the studies on colorectal cancer used different cell lines (HT-29 and T84 cells in [25] and HCT-116 cells in [28]), the discrepancy between the various studies remains to be resolved.

Theoretically, the use of Kv11.1 inhibitors into clinical practice may be far to be practicable, due to the correlated risk to develop fatal cardiac arrhythmia [41]. However, not all of the Kv11.1 inhibitors have the same proarrhythmic potential. For example, the Kv11.1 inhibitor fluoxetine, which have a moderated effect on electrocardiogram QT interval compared to other serotonin-selective reuptake inhibitor antidepressants, have been successfully used in glioblastoma therapy without obvious cardiotoxicity [42]. Another possibility might be to target hERG channels specifically in tumor cells using, for instance, a channel inhibitor conjugated to a monoclonal antibody directed against the tumor. Recently, a bispecific antibody targeting hERG and the β1 subunit of integrin receptors, which form a specific multimeric complex in tumor cells, was shown to reduce growth and vascularization in various solid tumor models, whereas it did not bind to the hERG1 channel in healthy tissues, including the heart [43]. Thus, the identification of molecules/strategies that allow a specific activity on tumors without eliciting off-tumor adverse effects is of crucial importance in order to overcome the complex safety-pharmacology problem.

Among voltage-gated channels, Kv10.1 (hEag1) was defined in ovarian cancer (OC) as a favorable marker to predict cancer sensitivity to cisplatin-based therapy. In vitro experiments demonstrated that OC cells’ resistance to cisplatin was strictly linked to Kv10.1 expression and NFkB cascade. Indeed, silencing of Kv10.1 in OC cells treated with cisplatin induced a significant decrease in NF-kB and Bcl-2 protein expression, sensitizing them to cisplatin-induced apoptosis [29]. The role of Kv10.1 was also studied in patients with hematological malignancies [30]. Channel expression was found in cancer cells in a subtype-dependent manner. Whereas the channel was missing in peripheral blood cells of healthy individuals or in patients affected by acute lymphoblastic leukemia, it was upregulated in part of the patients suffering from chronic myeloid leukemia, myelodysplastic syndrome, and acute myeloid leukemia (AML). In AML patients, expression was further dependent on disease subtype and patient age, and was predictive of a poor outcome, with higher relapse rates and a significantly shorter overall survival. Inhibition of Kv10.1 by the potassium channel blocker astemizole, the specific anti-Kv10.1 antibody mAb56, or siRNA strongly increased apoptosis in primary AML cells and cell lines treated with chemotherapeutic agents commonly used in AML therapy, including etoposide, cytarabine, and doxorubicin [30]. Furthermore, in 70% of AML cases, also Kv11.1 channels were upregulated and associated with an improved AML cell invasiveness, a higher rate of relapse, and a shorter survival [44]. Thus, the simultaneous inhibition of Kv10.1 and Kv11.1 by astemizole in AML cell types expressing both channels might be advantageous.

A strong correlation was found also between the expression levels of Kv1.1, Kv1.3 and Kv1.5 and sensitivity to chemotherapeutic drugs, but in the opposite direction [31,32]. The upregulation of these channels, linked to an increase of K^+^ current density, was associated with an enhanced sensitivity of several cancer cell lines to multiple chemotherapeutic drugs. It was shown that the K^+^ currents were involved in caspase activation in the early stage of apoptosis processes [45], while extracellular potassium modulated the apoptosis-associated tyrosine kinase activity in a Ca^2+^-dependent manner [46]. Han and collaborators demonstrated that the pro-apoptotic doxurubicin and 5-fluorouracil significantly increased the K^+^ current density in gastric cancer cells. The treatment of these cells with 4-aminopyridine or tetraethylammonium reduced doxurubicin-induced apoptosis. Silencing of Kv1.5 with siRNA reduced cell sensitivity to doxurubicin, 5-fluouracil, vincristine, and cisplatin, whereas Kv1.5 overexpression did the opposite, pinpointing Kv1.5 as an important player in gastric cancer chemosensitivity [31]. A similar relationship was also identified between Kv1.1 and Kv1.3 channel expression and susceptibility to death upon treatment with staurosporine and cisplatin in a panel of cancer cell lines [32]. It remains to clarify how K^+^ currents flowing through Kv1.x channels may have opposite effects to K^+^ currents flowing through hERG and EAG channels.

### 2.2. Calcium-Activated Potassium Channels

The KCa1.1 and KCa3.2 channels are the main calcium-activated potassium channels able to modulate directly chemotherapeutic-response in several cancers, including OC, glioblastoma, esophageal squamous carcinoma, melanoma, and osteosarcoma [33,34,47,48,49].

Focusing on drug resistance, it was demonstrated that the downregulation of KCa1.1 (KCNMA1), mediated by miR-31, enhanced resistance to cisplatin in OC cells, suggesting that KCa1.1 expression may represent a potential factor to predict cisplatin-response of OC patients. Indeed, analyzing OC tissues derived from a small cohort of patients, it was revealed that the levels of KCNMA1 mRNA were significantly reduced in drug resistant compared to sensitive cancers, which was consistent with the hypothesis that inhibition of KCa1.1 channels can lead to alterations in cisplatin sensitivity in OC patients [33].

The role of KCa1.1 channel in mediating chemoresistance was further investigated in glioblastoma, where cancer cells were able to survive in a heavy hypoxic microenvironment that exacerbates tumor aggressiveness [34]. In these cells, hypoxia induced a functional upregulation of KCa1.1 channel activity, facilitating migration and invasion processes and inducing chemoresistance to cisplatin. Thus, the use of KCa1.1 inhibitors prevented the hypoxia induced drug resistance, suggesting KCa1.1 as a potential therapeutic target in the treatment of glioblastoma [34].

Thus, as for other K^+^ channels, the role of KCa1.1 channels on chemoresistance may depend on tumor type.

### 2.3. Inwardly-Rectifying Potassium Channels

Among the different mechanisms responsible for chemoresistance studied in literature, a close link was reported between Kir2.1 channels and miRNA. The relevance of Kir2.1 (KCNJ2) channels in drug resistance was deeply investigated in small-cell lung cancer (SCLC), where these channels appear to be overexpressed [35]. The analysis of clinical SCLC tissues revealed that Kir2.1 overexpression was negatively associated with the chemotherapeutic response of SCLC patients, suggesting a potential predictive role of these channels in SCLC treatment. These results were confirmed by in vitro experiments showing that multidrug-resistant SCLC cell lines became more sensitive to doxurubicin, cisplatin and etoposide when Kir2.1 channels were knocked down, whereas Kir2.1 overexpression induced a desensitization of cells to these chemotherapeutic agents. Indeed, during chemotherapeutic treatment, Kir2.1 channels were able to control the cell cycle, inducing its arrest at the G0/G1 phase and inhibiting drug-induced apoptosis. Furthermore, these channels were widely regulated by several modulators that play critical roles in chemosensitivity. Among these, miR-7 is a known tumor suppressor, able to block cell growth and metastasis, promote apoptosis process and inhibit drug resistance. The downregulation of miR-7 in SCLC promoted Kir2.1 expression, which led to the activation of chemoresistance processes [35].

Altogether, these results shed new light on how K^+^ channels contribute to chemotherapy resistance, providing a potential therapeutic option for patients who frequently succumb to drug resistant disease. Some results, however, suggested opposite relationships, depending on the potassium channel subtype or, for the same channel, on the cancer type. Thus, more studies are needed to define specific strategies for each cancer types and implement a precision medicine strategy.

## 3. Sodium Channels

### 3.1. Voltage-Gated Sodium Channels

There is compelling evidence that voltage-gated sodium channels (VGSC) contribute to cell migration, invasion, and adhesion in a number of metastatic cancers [4,50,51,52]. In non-small cell lung carcinoma cell line, the upregulation of VGSC induced by epidermal growth factor (EGF) promotes cell invasion [53,54]. Since most of cancer cells have a depolarized membrane voltage (between −40 and −10 mV), most of the VGSC may be inactivated but there is still the possibility that a small sodium current may modify intracellular Na^+^ concentration, which in turn may modulate Na^+^ carrier activity [55]. Thus, pH and intracellular calcium regulation by voltage-gated sodium channels through coupling with membrane exchangers, especially in the invadopodia, have been suggested [52]. During metastasis, the VGSC are indeed involved in the process of invasion (for review see [55]). However, blockade of these channels by tetrodotoxin suppressed only partially this process, indicating that such a strategy may be insufficient to fully eradicate metastasis. In general, inhibition of VGSC may counteract metastasis but has less effect on cell proliferation. Metastasis requires the transition of cancer cells from the epithelial to the mesenchymal phenotype (EMT), which is characterized by enhanced resistance to apoptosis and drugs [56]. Inhibition of VGSC may block EMT but whether VGSC are directly implicated in drug resistance remains unclear [57,58]. First suggestion of a possible involvement of VGSC in drug resistance was provided by experiments performed in human leukemia cells [59]. These researchers showed with the patch-clamp technique that multidrug-resistant cell lines (resistant to anthracycline antibiotics and Vinca alkaloids) exhibit tetrodotoxin-sensitive, voltage-activated sodium currents, whereas drug-sensitive cell line counterpart did not. However, tetrodotoxin was not able to rescue sensitivity to doxurubicin and vincristine, challenging the relationship between drug resistance and VGSC [59]. A very recent study suggested that lidocaine sensitizes ovarian cancer cells to cisplatin by blocking Nav1.5 sodium channel subtype. In OC cells treated with 10 μM of cisplatin combined with lidocaine (5 mM), cell viability was decreased by 40% compared with OC cells treated with cisplatin alone [60]. Thus, sodium channel inhibition in OC did not counter chemoresistance completely, but at least in part potentiated the effect of cisplatin. Such an effect might rely on the inactivation of FAK/Paxillin signaling pathway and induction of apoptosis [60]. However, another study suggested that Nav1.5 enhances 5-Fluouracil stimulated apoptosis in colorectal cancer cells [61]. Thus, further studies are required to verify the role of VGSC in cancer cell drug resistance.

### 3.2. ENaC/Deg Ion Channels

The ENaC/Deg superfamily of ion channels mainly conduct sodium ions, including the epithelial sodium channel (ENac) and the acid-sensing ion channels (ASIC). A few studies suggested the possible involvement of these channels in cancer [62,63,64,65,66,67,68,69,70]. ENac is well known for its role in sodium ion reabsorption in epithelia, but it may also contribute to vascular smooth muscle cell migration, suggesting a role in angiogenesis [62]. In human liver hepatocellular carcinoma HepG2 cells, hypertonic stress increased Na conductance and stimulated cell proliferation, while inhibition of ENaC channel with small molecules or siRNA strongly decreased the rate of cell proliferation and promoted apoptosis [63]. Yet, in breast cancer, enhanced ENaC levels and activity were positively correlated with prognosis and were shown to reduce cell proliferation [64]. No data are available regarding the role of ENac in cancer cell drug sensitivity.

**Figure 1:**
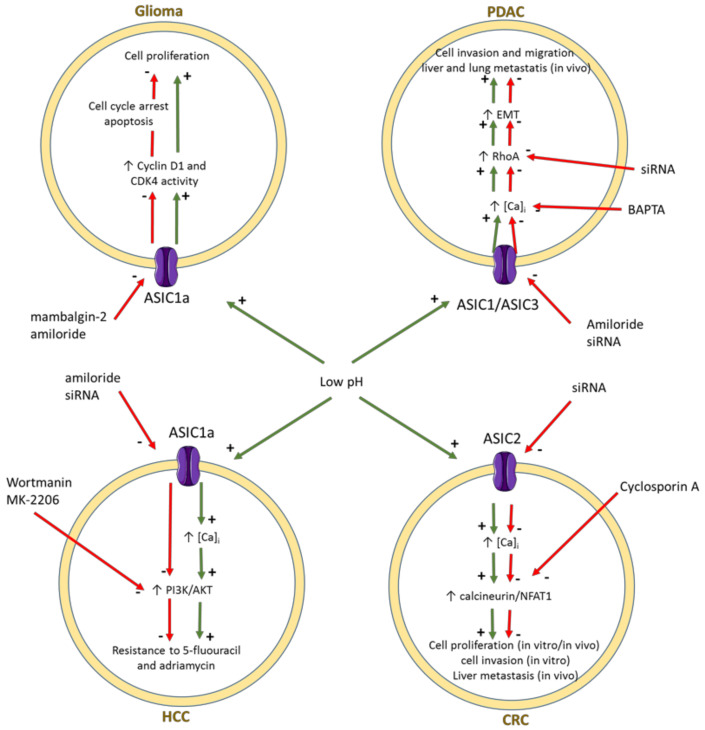
Schematic representation of signaling pathways involved in the effects of ASIC channels in glioma [67], pancreatic ductal adenocarcinoma (PDAC) [69], hepatocellular cancer (HCC) [70], and colorectal cancer (CRC) [68]. The activation and inhibition pathways are represented by green and red arrows, respectively. EMT: epithelial-mesenchymal transition.

The sensitivity to acidic pH makes ASIC of particular relevance for the tumor cell fate in the tumor microenvironment. Inhibition of ASIC1 channels blocks cell migration and induces cell cycle arrest and apoptosis in glioblastoma [65,66,67]. In colorectal cancer, ASIC2 was involved in invasion and metastasis promoted by acidosis through activation of the calcineurin/NFAT1 axis [68]. Both ASIC1 and ASIC3 channels were shown to regulate the acidity-induced epithelial mesenchymal transition, likely through the intracellular calcium-RhoA signaling pathway, in pancreatic ductal adenocarcinoma cell lines [69]. Interestingly, inhibition of ASIC1 was able to enhance sensitivity to 5-fluorouracil in hepatocarcinoma cell lines and a number of experiments suggested that ASIC may mediate drug resistance via the calcium/PI3K/AKT signaling pathway [70]. The involvement of ASIC channels in various cancer cell types is resumed in Figure 1. It should be noted that the effects of ASIC activation reported up to now might rely at least partially on calcium ion permeability [69,70,71].

## 4. Calcium Channels

Calcium signaling represents a critical regulator of a plethora of cellular processes, many of which intersect with those important in cancer proliferation, progression and sensitivity to chemotherapeutics [72,73]. The involvement of calcium signals in chemoresistance has been widely studied in the last years, when a growing number of studies demonstrated a tight link between Ca^2+^ channel expression and chemosensitivity in resistant cancer cells. Given the intrinsic link between increases in cytoplasmic calcium concentration and cell death [74], it is not surprising that calcium channels have been investigated as potential ways to promote the death of cancer cells. The main calcium channels that may be involved in this process include voltage-gated calcium channels (VGCC); store-operated channels and transient receptor potential channels (TRPs).

### 4.1. Voltage-Gated Calcium Channels

The voltage-gated calcium channels (VGCC) are the most selective channels for calcium ions, activated by plasma membrane depolarization. According to sequence homology and functional properties, VGCC can be classified into three different families: high-voltage-activated L-type channels (Cav1), high-voltage-activated P/Q-type, N-type, and R-type channels (Cav2), and low-voltage-activated T-type channels (Cav3) [75].

The T-type and L-type Ca^2+^ channels are considered the two main kinds of VGCC aberrantly expressed in different cancers and involved in chemoresistance process [76].

The role of L-type calcium channel in drug resistance was studied in in vivo and in vitro models of diffuse large B-cell lymphoma (DLBCL) [77]. Patients with DLBCL are usually treated with a standard chemotherapy (cyclophosphamide, vincristine, doxorubicin, prednisone) in addition with rituximab. Although the combined therapy significantly improved overall survival, one third of DLBCL patients succumb to the disease due in part to rituximab resistance. Besides cell-mediated and complement-mediated cytotoxicity, rituximab was shown to activate directly apoptosis [78]. In B cells, when crosslinking two CD20 molecules, CD20 caused CD20 redistribution to plasma membrane lipid rafts, and increased calcium influx that, in turn, activated apoptotic pathway [79]. Although it was proposed that CD20 might mediate or modulate store-operated calcium entry in B cells [80], a role for the L-type Cav1.2 channel was also recently suggested [77].

In DLBLC rituximab-resistant patients, the mRNA and protein expression of Cav1.2 was lower compared with rituximab-sensitive patients, suggesting a role of Cav1.2 in enhancing clinical efficacy of rituximab. Biochemical experiments performed in DLBLC cell lines and in xenograft mouse models demonstrated that loss of Cav1.2 expression led to lower CD20 stability, leading to reduced rituximab-induced cell death. The combined use of rituximab with Bay K8644, an agonist of L-type calcium channels, significantly increased apoptosis processes in DLBCL cell lines and markedly reduced tumor volume and weight in vivo in DLBCL patient-derived xenografted mice [77]. Thus, the modulation of L-type calcium channel may increase the clinical efficacy of rituximab, suggesting a new potential approach to raise therapeutic efficacy for DLBCL patients. However, the risk of side effects associated with the use of calcium channel agonists, including hypertension, will require carefully consideration.

Regarding T-type channels, the role of Cav3.1 and Cav3.2 has been widely investigated in several cancer types, where they were shown to regulate different signaling pathways involved in cancer cell proliferation, survival, and invasiveness [81,82]. More recently, an increasing number of studies have shown a clear link between T-type Ca^2+^ channel expression and sensitivity to therapeutic drugs. In glioblastoma, a positive association was identified between the overexpression of Cav3.1 and chemotherapeutic resistance to the alkylating agent temozolomide. One concern about the use of temozolomide regarded the activation of autophagy as a proadaptive cellular response to therapy, which may confer drug resistance to cancer cells. The study of the autophagic status of temozolomide-resistant glioblastoma cells suggested that the combined application of temozolomide and autophagy inhibitors synergistically increased cytotoxicity [83,84,85]. In an in vitro model of temozolomide-resistant glioblastoma, the activation of autophagy was accompanied by the overexpression of Cav3.1, while the knockdown of these channels led to deficient autophagy. These findings identified Cav3.1 calcium channels as a molecular target to regulate autophagy and prevent chemotherapeutic resistance in glioblastoma [86]. A strong correlation between autophagy and T-type Ca^2+^ channels was also evidenced in cutaneous melanoma, in which it was demonstrated that the overexpression of Cav3.1 and the increase of basal autophagy induced the arising intrinsic mechanism of BRAF inhibitor resistance [87].

A tight association between T-type Ca^2+^ channels and the expression of antiapoptotic proteins was revealed in platinum-resistance ovarian cancer cells. In this respect, the inhibition of T-type Ca^2+^ channels with mibefradil or siRNA induced proapoptotic responses through reduced expression of the antiapoptotic gene *survivin*, suggesting that intervention on this pathway may be an effective approach to increase OC response to therapy [6,88].

### 4.2. Store-Operated Calcium Channels

Store operated calcium entry (SOCE) is one of the main pathways of calcium entry into mammalian cells, which typically allows calcium influx through the plasma membrane subsequent to endoplasmic reticulum depletion. The two major participants in SOCE comprise the plasma membrane channel Orai1 and the endoplasmic reticulum (ER) channel STIM1 [89].

STIM proteins (STIM1 and STIM2) are Ca^2+^ sensors located on the endoplasmic reticulum (ER) membrane and are essential for Ca^2+^-store-depletion-triggered Ca^2+^ influx, whereas ORAI proteins are plasma membrane (PM) channels that can be gated by STIMs for Ca^2+^ entry during SOCE [90,91]. The N-terminus and C-terminus of ORAI intracellular sites are essential for the interaction with STIM1 and the opening of the ORAI channel [92]. When STIM1 residing in the ER lumen senses a Ca^2+^ decrease in the ER, it initiates conformational changes and oligomerization, leading to its activation. Activated STIM1 multimerizes and migrates toward the PM, where it interacts with the intracellular regions of ORAI, resulting in ORAI activation and Ca^2+^ influx [90,91].

When STIM1 senses loss of ER Ca^2+^, it unfurls domains that interact with Orai1 Ca^2+^ channels allowing calcium entry, which regulates a variety of cellular processes and functions such as cell proliferation [92], skeletal muscle development and contraction [93], and smooth cell migration [94]. Growing evidence supports the hypothesis that STIM1 and Orai1 are implicated in a number of pathological processes in cancer, including human glioblastoma invasion [95], acute myeloid leukemia cell migration, and breast tumor cell migration [96].

Previous studies have reported conflicting results about the role of these channels in apoptosis regulation and chemoresistance. Some reports suggested that Orai1 and STIM1 play a pro-survival anti-apoptotic role, whereas others suggested their contribution to apoptosis induction.

For instance, it was reported that pancreatic adenocarcinoma cell lines treated with 5-Fluorouracil and gemcitabine showed an upregulation of Orai1 and STIM1, increasing SOCE and promoting cell survival. These results emphasized the anti-apoptotic role of Orai1 and STIM1 and revealed that chemotherapy may have calcium-dependent effects unrelated to the primary DNA-targeting mechanisms of their action [97]. Thus, the downregulation of these channels makes cells more sensitive to apoptosis induction, overcoming chemoresistance.

In line with this, it was demonstrated that the expression of Orai1 and STIM1 was significantly higher in cisplatin-resistant A2780cis ovarian cancer (OC) cells compared to cisplatin-sensitive A2780 OC cells [98]. The overexpression of Orai1/STIM1 was paralleled by the enhancement of Akt1, a serine/threonine kinase implicated in cancer cell proliferation, inhibition of apoptosis, and establishment of therapy resistance [99]. Pharmacological inhibition of either Akt or Orai1 increased cisplatin-induced apoptosis of A2780 cisplatin-resistant OC cells, abrogating the differences in cisplatin sensitivity between them and A2780 non-resistant cells [98].

Similarly, STIM overexpression in osteosarcoma cells protected them from undergoing cisplatin-induced apoptosis. Previous studies reported that cisplatin might induce ER stress, a chronic perturbation characterized by an accumulation of aberrant proteins, which disturbs the balance of the protein folding capacity of the ER and leads to apoptosis [100]. This process is closely correlated to STIM and SOCE. Indeed, STIM1 knockdown sensitizes cisplatin-resistant osteosarcoma cells to cisplatin via enhancing ER stress-mediated apoptosis [101].

Whilst Orai1 was widely studied in chemoresistance, recent work had established a strong link between Orai3 and resistance in breast cancer. Induced overexpression of Orai3 in T47D breast cancer cells led to resistance to cisplatin, 5-fluouracil and paclitaxel [102]. The mechanism by which Orai3 induced chemoresistance was linked to the increase of SOCE and the consequent downregulation of the tumor suppressor protein p53 via PI3K activation [102]. All of these studies demonstrated that overexpression of STIM1 and Orai1 conferred chemoresistance to a number of cancers.

In contrast with this, it was proposed that low Orai1 expression in prostate cancer cells might contribute to the establishment of an apoptosis-resistant phenotype, irrespective of the apoptosis-inducing stimulus [103]. These discrepancies could be explained by different clues. First, different cell lines were used in these studies, each differentially expressing ORAI and STIM proteins. Second, it is known that both Orai1 and STIM1 may exert SOCE independent functions. For instance, it was reported that Orai1 could stimulate mammary tumorigenesis through store- and STIM1-independent pathway but involving the secretory pathway Ca^2+^-ATPase, SPCA2 [104].

Considering such a complex regulatory network and the role of Orai1/STIM1 in both pro-survival and pro-apoptotic processes, the resistance of each cell line could differ depending on the signaling pathway activated. Thus, the comprehension of ORAI and STIM channels’ role in the resistance to cancer therapy in different types of cancers is now a priority. The diversity of resistance pathways associated with cytotoxic and emerging molecularly targeted therapies provides an array of avenues for investigation.

### 4.3. Transient Receptor Potential (TRP) Channels

Transient receptor potential (TRP) channels are non-selective and calcium-permeable ion channels divided into six subfamilies including TRPC (Canonical), TRPM (Melastatin), TRPV (Vanilloid), TRPA (Ankyrin), TRPP (Polycystic), and TRPML (Mucolipin) [105].

Under different stimuli, TRP channels induce Ca^2+^ influx resulting in a transient intracellular calcium increase and in a rapid modulation of the calcium signaling pathways.

Over the last decade, TRP channels have gained attention in the field of cancer therapy. It has been found that changes in expression and function were strictly correlated to resistance or sensitivity of cancer cells to apoptotic-induced cell death, resulting in cancer-promoting effects or resistance to chemotherapy treatments.

The TRPC subfamily consists of seven members (TRPC1-7) that function as non-selective cationic channels permeable to Ca^2+^, Na^+^, and K^+^ [106]. The role of TRPC1 in cancer has been widely investigated in a number of studies, showing that its overexpression was related with poorly differentiated tumors and higher cell proliferation, motility, and hypoxia-induced autophagy [107,108]. The role of TRPC1 channels was also investigated in cancer therapy resistance. In vitro experiments revealed that cisplatin and carboplatin-resistant OC cells showed a marked decrease in TRPC1 mRNA levels, compared with their sensitive counterparts [109]. TRPC1 contributes to cisplatin resistance in OC through a direct interaction with two proteins involved in ERK-mediated autophagy (PIK3C3 and SPARCL1) [109].

Autophagy can be also mediated by TRPC5, a Ca^2+^-permeable channel involved in cancer angiogenesis and chemoresistance [108]. This latter may be involved in chemoresistance through various mechanisms. Calcium entry mediated by TRPC5 overexpression in drug-resistant MCF-7/ADM cells induced an upregulation of P-glycoprotein, pumping cytotoxic drugs from tumor cells and maintaining chemoresistance in drug-resistant MCF-7/ADM cells [110]. In line with this, TRPC5 was demonstrated to be essential for the induction of ABCB1-mediated resistance to 5-fluouracil in colorectal cancer cells. The increase of intracellular calcium level triggered by TRPC5 induced the translocation of β-catenin into the nucleus and subsequently up-regulated expression of the Wnt target genes cyclin D1 and ABCB1 [111]. More recently, autophagy was identified as a novel mechanism of TRPC5 to induce doxurubicin resistance in breast cancer cells. Zhang and colleagues demonstrated that there was a positive correlation between TRPC5 and the autophagy-associated protein LC3. Using pharmacological inhibition or gene silencing, it was shown that the cytoprotective autophagy mediated by TRPC5 during doxurubicin treatment depends on the CaMKKβ/AMPKα/mTOR pathway. Moreover, doxurubicin-resistant MCF-7/ADM cells maintained a high basal level of autophagy, while the silencing of TRPC5 and inhibition of autophagy counteracted the resistance to doxurubicin [112]. Thus, these findings revealed a novel role of TRPC5 as an inducer of autophagy, and suggested that this channel may be a target for the reversal of clinical chemoresistance.

The TRPC6 channel is overexpressed in hepatocellular carcinoma (HCC) cells, where it may promote the sustained accumulation of intracellular calcium that influences multidrug resistance processes [113]. The intracellular elevation of calcium mediates mechanisms of the epithelial-mesenchymal transition, Hif1-α signaling, and DNA damage repair, thereby leading to chemoresistance. In line with this, the inhibition of TRPC6 with its specific blocker SKF-96365 or with siRNA reverted doxorubicin-induced epithelial-mesenchymal transition, hypoxia-induced Hif1- α expression and inhibit DNA damage, thereby attenuating drug resistance in hepatocellular carcinoma cells [113].

Concerning the TRPM family, the main channels involved in cancer therapy resistance include TRPM2 and TRPM8. TRPM2 is widely considered as a potential therapeutic target, due to its crucial role in sustaining mitochondrial function, cell proliferation, and tumor metastasis in several types of cancers [114]. A number of studies revealed that inhibition or genetic deletion of TRPM2 significantly enhances anti-cancer drug cytotoxicity in neuroblastoma, breast and gastric cancers [115,116,117]. In gastric cancer, the knockdown of TRPM2 inhibited autophagy process, thus sensitizing gastric cancer cells to paclitaxel and doxorubicin [117].

The involvement of TRPM8 in chemoresistance was suggested in pancreatic cancer cells and tissues, where these channels appeared to be overexpressed [118]. Silencing of TRPM8 suppressed the expression of protein involved in gemcitabine resistance, such as P-glycoprotein, MRP2, and LRP multi-drug associated proteins. Furthermore, TRPM8 knockdown significantly increased the expression of gemcitabine metabolism-related proteins involved in the cellular uptake of the drug and the expression of apoptosis-related proteins, suggesting that TRPM8 not only affected gemcitabine resistance but also the apoptosis of prostatic cancer cells [118].

## 5. Chloride Channels

### 5.1. Putative Chloride Channels from the CLIC Protein Family

CLIC proteins (CLIC stands for chloride intracellular channel) have been proposed to be anion channels localized in intracellular membranes. The founding member of the CLIC family, called p64, was cloned in 1989 by affinity to IAA94, a chloride channel blocker [119]. However, as discussed in detail by Jentsch and collaborators [120], the association of the cloned protein with the originally described chloride channel activity was dubious, seriously questioning any channel property of p64. By homology, other CLIC proteins have been described with at most one putative transmembrane domain [121]. Further studies revealed that CLIC proteins are actually soluble cytosolic proteins with the fold of omega class glutathione transferases [120]. The most cited evidence relating CLIC proteins to ion transport is that some CLIC proteins have been reported to be able to interact with lipid membranes and to induce channel activity in reconstituted systems [121]. However, the physiological relevance of this phenomenon remains unclear, and convincing evidence that CLIC proteins are structural parts of anion channels is lacking. Thus, in the present review focused on ion channels, we will not discuss the involvement of CLIC proteins in cancer [122], which is most likely related to their enzymatic activity.

### 5.2. Calcium-Activated Chloride Channels (CaCC)

The term TMEM protein was coined for predicted transmembrane proteins with unknown function at the time of their sequence identification. Indeed, TMEM members are expressed in many cell types and are involved in various physiological processes generally performed by membrane proteins (receptors, anchorage and structural proteins, transporters, enzymes). However, due to difficulties in protein extraction and purification, the role of many members is still poorly characterized [123].

Many members of the TMEM family are implicated in cancer. In this context, they may act as tumor suppressors or as oncogenes, being involved in tumor growth and generally associated with a poor patient prognosis. A relevant role has been assumed by several members as prognostic biomarker [124].

We will concentrate on TMEM16A, which is a Ca^2+^-activated chloride channel that has been implicated in cancer [125,126,127]. Interestingly, TMEM16 proteins are part of a gene family that comprises ten members with high sequence identity [128]. However only TMEM16A and TMEM16B encode ion channels, while the other eight TMEM16 members are probably plasma membrane or intracellular lipid scramblases (i.e., proteins that swap lipids between the outer and inner leaflet of membranes), resulting, for example, in the exposure of phosphatidylserine lipids to the outside of platelets, an important event in blood clotting [129]. At least two TMEM scramblases (TMEM16E and TMEM16F) are associated with non-selective ion transport activity [128,130]. The physiological role of this transport is however unclear.

TMEM16A (also called ANO1 from ‘Anoctamin 1’ or DOG1 from being ‘discovered on GIST-1’) is the first identified member of the calcium activated chloride channel (CaCC) family. Structurally, it is organized as a dimer, with each subunit composed of ten conserved transmembrane domains, and intracellular N and C termini [131,132]. It has been shown to have a role in Cl^−^ homeostasis of various epithelial tissues such as vascular endothelia, gastrointestinal epithelia [133,134], in trans-epithelial secretion [135,136]. Moreover, TMEM16A has impact in exocrine gland physiology, smooth muscle contraction, neuronal excitability, and sensory transduction [137,138].

A large number of studies has recognized that TMEM16A is aberrantly expressed in numerous malignancies such as human neck squamous cell cancer (HNSCC), breast cancer, gastrointestinal stromal cancer, and prostate cancer [139]. The most recurrently observed dysregulation is a significant overexpression of the TMEM16A gene that probably represents the general mechanism relating TMEM16A with carcinogenesis [140]. The channel overexpression in turn affects a variety of biological functions, such as cell proliferation, migration and invasion and resistance to apoptosis. Notably, an unusual heterogeneity of the molecular mechanisms at the origin of TMEM16A oncogenic over-expression has also emerged. In several cases, cancers are correlated with amplification of the 11q13 locus, where TMEM16A maps, which determines a high proliferation effect probably due to the activity of other genes, contained in the amplicon [141]. In some other cases, the epigenetic level seems to be implicated. Indeed, hypermethylation of the TMEM16A promoter has been reported to decrease the expression level of the gene itself [142], while it is likely that hypomethylation affects expression in the opposite way. In addition, TMEM16A mRNA is a target of several micro RNAs that are down regulated in two types of cancers (gastric and colorectal) characterized by high expression of the channel [143,144]. Finally, the transport activity of TMEM16A in a wide range of different cell types implies its participation in a high number of downstream signaling pathways that channel dysregulation may affect [128]. Interestingly, TMEM16A regulates the epithelial-mesenchymal transition interacting with radixin and has been shown to switch the tumor phenotype from proliferative to metastatic [142].

A significant fraction of human HNSCC cases show up-regulation of ATP7B, a P-type ATPase engaged in transferring copper ions out of the cells, and over-expression of TMEM16A ion channels as well [145]. Similar to many other solid tumors, HNSCC is treated with platinum-based drugs, even though a significant resistance largely reduces patient survival rate to 40–50%. Several pieces of evidence from various groups link ATP7B expression to cisplatin resistance. Usually located in the trans Golgi network, the transporter has been shown to relocate in resistant cells of ovarian cancers, being accumulated in the peripheral compartment near the cell surface, where it can more easily introduce platinum into lysosomes or related vesicles ready to be expelled from the cells [146]. Starting from the strong correlation of ATP7B and TMEM16A expression observed in human HNSCC tumor samples isolated from 13 patients, Vyas and collaborators went on investigating transfected cells and concluded that TMEM16A protein overexpression in turn determines ATP7B mRNA levels, and thus cisplatin cell sensitivity, probably through a mechanism involving oxidative stress [145]. Epidermal growth factor receptor (EGFR) is implicated in HNSCC pathogenesis. Indeed, its overexpression has been ubiquitously observed in this type of cancer and has been correlated with decreased patient survival [147,148]. Nevertheless, only a small portion of patients responds to EGFR directed therapy. Indeed, the monotherapy with gefitinib, a tyrosine kinase inhibitor, or with the EGFR antibody cetuximab is poorly efficacious [149]. Bill and collaborators found that in HNSCC cells TMEM16A interacts with EGFR, forming a stable functional complex, which promotes cell proliferation but is independent of the chloride transport activity of the channel, rather requiring the physical interaction of TMEM16A with a transmembrane domain of EGFR [150]. Interestingly, HNSCC cells highly expressing the complex showed enhanced sensitivity to gefitinib, probably since TMEM16A expression acts positively on EGFR signaling and renders HNSCC cells more sensitive to EGFR inhibition. In conclusion, co-targeting of the TMEM16A-EGFR complex could improve the clinical efficacy of therapies reducing HNSSC monotherapy resistance. Unfortunately, the in vivo validation of these data is difficult due to the lack of TMEM16A specific inhibitors [150]. Similarly, TMEM16A inhibition was found to improve the efficacy of EGFR targeted therapy in HNSCC by increasing the sensitivity to cetuximab [151].

Apoptosis suppression is frequently adopted by cancer cells to resist to chemotherapy. However, the mechanisms implicated still need to be investigated. In vitro and in vivo studies from Godse and collaborators demonstrated that high expression of TMEM16 limits apoptosis in HNSCC cells, both in general and, most importantly, in response to cisplatin that has a strong pro-apoptotic activity. This effect was proposed to be mediated by a reduction of the pro-apoptotic BIM protein, as also confirmed by the analysis of a panel of 41 human HNSCC samples [152]. According to these data, it is possible that inhibition of TMEM16A or of its downstream pathways such as ERK1/2 can overcome the cisplatin resistance encountered in highly TMEM16A expressing cancers [153].

Overall, the data described above converge in attributing to TMEM16A an important and multifaceted role in the resistance of HNSCC to therapy.

TMEM16A activity in cancer drug resistance has been explored in other cancer types as well. In BCA cell lines with HER2 amplification, the development of resistance to the HER2 inhibitor trastuzumab was correlated with an increase in TMEM16A expression, while the combined suppression of HER2 and TMEM16A improved the response to the biologic therapy [151], thus offering a clinical advantage. Indeed, according to these authors, TMEM16A, by activating HER family proteins through phosphorylation, would decrease the response to therapy, while TMEM16A silencing would reduce cell viability and tumor growth. The described effects could be mediated either by Cl^-^ fluxes or by TMEM16A protein abundance that could directly interact with and thus modulate HER protein stability [151]. Further experiments are necessary to resolve the matter. A similar connection was reported by Fujimoto and collaborators: working on YMB-1 Her-positive BCA cells resistant to trastuzumab, they found that TMEM16A blockers sensibly suppressed cell viability, thus suggesting that sensitivity to HER2 addressed therapy, approved for treatment of metastatic breast and cancer therapy, can be modulated by the presence of TMEM16A [153].

In summary, uncontrolled expression of TMEM16A has a large impact in different types of cancers, but the absence of specific and potent inhibitors has hitherto prevented its targeting in clinical applications.

### 5.3. Volume Regulated Anion Channels (VRAC)

In 1988, two groups independently discovered the presence of anionic currents activated by exposing lymphocytes or epithelial cells to a hypotonic extracellular solution [154,155]. Afterwards, similar anion currents activated by hypotonicity have been observed in practically all vertebrate cells [156]. The biophysical properties of these currents differed in cell types of different origins, and for this reason, various names have been used for them. Here, we will stick to the most common nomenclature of volume-regulated anion channels (VRACs) [157]. Physiological functions attributed to VRAC include involvement in volume regulation [158], proliferation [159], migration [160], and apoptotic volume decrease [161], release of excitatory amino acids and ATP [162,163], among several others [156]. Before their molecular identification, VRACs have not been implicated in resistance to cancer drug resistance.

Only in 2014 have the genes been identified that underlie VRACs: members of the Leucine Rich Repeat Containing 8 family, comprising LRRC8A, -B, -C, -D, and -E [164,165]. Voss and collaborators demonstrated that VRACs are heteromers composed of the obligatory LRRC8A subunit and at least one other subunit among LRRC8B, -C, -D, or -E [165]. The LRRC8 proteins are composed of an N-terminal transmembrane domain harboring four transmembrane helices with sequence homology to pannexins and a C-terminal leucine rich-repeat domain with about 17 leucine-rich repeats [166]. The hexameric architecture, predicted by Abascal and Zardoya [166], was beautifully confirmed by a series of cryo-EM structures of (non-physiological) LRRC8A homomers [167,168,169,170]. Of importance for the possible role of VRACs in drug resistance, one of the structures showed the VRAC inhibitor bound to the extracellular narrowest part of the pore [170].

Different LRRC8 subunit combinations have different biophysical properties, such as speed and degree of inactivation at positive voltages [165,171,172]. Of particular importance are the various permeability properties exhibited by different combinations. For example, LRRC8A/LRRC8D channels have an increased permeability for larger molecules such as taurine and glycine compared to LRRC8A/LRRC8C or LRRC8A/LRRC8E subunit combinations [173]. However, electrophysiological measurements require the substrates to be charged, such that taurine and glycine permeability was measured at alkaline pH [173,174]. Using radioactive flux measurements at neutral pH, Lutter and collaborators found that LRRC8 channels are permeable for osmolytes taurine, myo-inositol, glutamate, aspartate, GABA and D-serine [175]. Importantly, LRRC8D was particularly important for permeation of overall neutral molecules such as myo-inositol, taurine and GABA and even allowed transport of positively charged lysine. Negatively charged amino acids such as aspartate were also transported by LRRC8A/LRRC8E heteromers [175], as seen also by electrophysiology [173]. Addition of further subunits (LRRC8B, -C) altered transport selectivity [175], indicating a highly complex situation in vivo.

Many experiments linked VRAC to the cell cycle and to cell proliferation [176]. Indeed, blockers of VRAC slowed down or arrested proliferation of endothelial cells, neuroblastoma cells, cervical cells, or hepatocytes [176]. On the other hand, cell shrinkage due to VRAC activation is essential for apoptosis. This role was confirmed from VRAC down regulation found in several types of drug-resistant cancer cells that show a decreased propensity for apoptosis [177]. In particular, several studies indicated an important role of VRAC in cisplatin resistance. For example, blocking VRAC by Cl^-^ channel inhibitors (SITS, DIDS) increased resistance to cisplatin [178]. Furthermore, cisplatin resistant KCP-4 cells, which lack VRAC activity, showed decreased resistance when VRAC activity was restored [179]. Additionally, Cl^-^ channel inhibitors are able to prevent the early shrinkage that precedes apoptosis [161], and to decrease cisplatin-stimulated caspase 3 activity [180]. Similarly, in human lung adenocarcinoma cells (A549 cells), cisplatin resistance was negatively correlated with DIDS dependent Cl^-^ channel activity [181].

In agreement with the reports of VRAC involvement in cisplatin resistance, Planells-Cases and collaborators discovered in an unbiased genetic screen that the inactivation of LRRC8A or of LRRC8D conferred partial cisplatin and carboplatin resistance in haploid KBM7 cells [182]. In addition, using a similar gene trap screen, Lee and collaborators found that inactivation of LRRC8D conferred resistance to the antibiotic blasticidin [183]. The LRRC8 proteins appeared to play a dual role; they mediate cisplatin/carboplatin uptake and they facilitate apoptosis. Earlier studies suggested that cisplatin/carboplatin uptake involves two components: about half enter cells by diffusion through the plasma membrane, and the other half via a protein component, possibly a channel [184]. In agreement with this, uptake experiments suggested that cisplatin/carboplatin could enter cells trough LRRC8A/8D heteromers [182]. Additionally, patients with a low LRRC8D gene expression in their ovarian cancers displayed reduced survival [182]. Experiments in *Xenopus* oocytes confirmed permeability of LRRC8A/8D, but also of LRRC8A/8E heteromers [185]. Incubation with cisplatin dramatically activated currents for both subunit combinations, confirming that VRAC channels provide an uptake pathway for cisplatin and that intracellular cisplatin accumulation strongly activates the channels [185].

These experiments render in particular LRRC8D containing heteromeric VRAC channels as a promising target to decrease cisplatin and carboplatin resistance.

## 6. Conclusions

**Figure 2:**
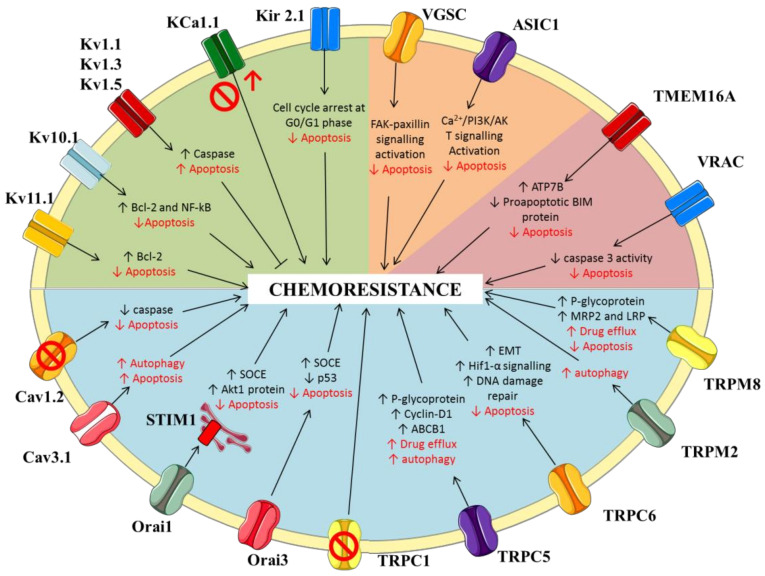
Ion channels possibly involved in drug resistance. Schematic representation of the main ion channels possibly involved in drug resistance and the molecular pathways through which these proteins may modulate chemosensitivity (K^+^ channels, green area of the cell; calcium channels: blue area; sodium channels: orange area; and chloride channels: pink area). EMT: epithelial-mesenchymal transition; SOCE: store-operated Ca^2+^ entry.

In conclusion, there is growing evidence that ion channels may contribute to chemoresistance in cancer. This is especially true for potassium, calcium, and chloride channels (Figure 2). More studies are needed to confirm a role for sodium channels. The demonstration of cause-effect relationship between ion channels and drug resistance requires accurate experiments of channel silencing by using both small inhibitors and siRNA. When possible, in vitro experiments on cell lines should be verified on human bioptic cancer tissues. In vivo experiments in animal models are also very important to confirm effects of ion channel modulation on tumor growth, metastasis, and chemosensitivity. Results should always be carefully interpreted, as a number of ion channel blockers have been shown to increase multidrug resistance by acting directly on ABC carriers and independently of their action on ion channels; such compounds include verapamil, quinidine, and A-803467 [186].

An important goal of future studies will be to define further the molecular mechanisms underlying an increase or decrease of resistance to specific chemotherapeutic compounds by enhanced or decreased ion channel expression. For VRAC channels, a direct contribution via facilitation of permeation of chemotherapeutics through the plasma membrane has been suggested [182]. For most other channels, delineating molecular mechanisms is a complex task since channel activity directly and indirectly impinges on various cellular factors including membrane potential, concentration of intracellular ions and pH, osmolarity, ionic strength, and others, with the intracellular calcium concentration being likely one of the most crucial parameters. In addition, mechanisms not directly linked to functional ion channel activity (e.g., by providing a binding partner for other proteins or involvement in cytoskeletal architecture) cannot be excluded. Finally, the effects of ion channel and the underlying molecular mechanisms can differ between tumors, and it might vary further between tumor stages. Interindividual variations might also occur, and the development of specific diagnostic markers might be required to personalize treatment.

Once their role in drug resistance is well established, ion channels represent attractive pharmacological targets. The advantages displayed by ion channels include their plasma membrane localization, the possibility to develop specific monoclonal antibodies, the number of marketed ion channel drugs available for repurposing, and the high-throughput screening platforms available for drug discovery. It should be noted, however, that anti-channel monoclonal antibodies are only in the early stages of development [187,188,189]. In addition, most available drugs are ion channel blockers, and the strategies are rather limited when an increased expression/activity is required. One critical limitation may be that the expression of ion channels is not limited to cancer cells, raising treatment safety concerns. Thus, searching for drugs acting on ion-channel splice variants specifically expressed in cancer cells may be of particular relevance [43,190].

It should be noted that, in many experiments, modulation of ion channels had only partial effects on cancer cell behavior and chemoresistance, suggesting that such a strategy may integrate existing combined therapy to enhance success rate.

Based on the relevance and growing interest of the topic, it is expected that drugs targeting ion channels will soon enter the arsenal of personalized adjuvant therapies to reduce the risk of chemoresistance development.

## Figures and Tables

**Table 1:** Summary of main effects of potassium channels in cancer cell chemoresistance.

K^+^ Channel	Cancer Models	Main Results	Relationship with Chemosensitivity	Ref.
Kv11.1 (hERG)	Various cancer cell lines (colorectal, breast, lung)	Positive correlation between level of expression and sensitivity to vincristine, camptothecin, or paclitaxel. Overexpression of Kv11.1 increased chemosensitivity.	More expression → more sensitivity	[25]
	gastric cancer (in vitro cell lines and in vivo mouse model)	Cisplatin increased Kv11.1 expression; Silencing Kv11.1 with siRNA decreased sensitivity to cisplatin by interfering with Bcl-2-dependent apoptosis.	Less expression/activity → less sensitivity	[26]
	Acute lymphoblastic leukemia (cell lines and primary cell culture, and in vivo mouse model)	Kv11.1 inhibition by blockers and siRNA reduced bone marrow mesenchymal cell-induced resistance of leukemic cells to doxorubicin, prednisone, or methotrexate.	Less expression/activity → more sensitivity	[27]
	colorectal cancer (in vitro cell lines and in vivo mouse model)	Increased expression/activity in cisplatin-resistant cell line; Inhibition of Kv11.1 increased cisplatin uptake and ciplastin-induced apoptosis in vitro, and overcome cisplatin resistance in vivo.	Less expression/activity → more sensitivity	[28]
Kv10.1 (hEag1)	Ovarian cancer (OC) (patient biopsies and cell lines)	Overall survival longer in cisplatin-treated OC patients with lower Kv10.1 expression; Silencing of Kv10.1 increased sensitivity to cisplatin by interfering with NFkB/Bcl-2 dependent apoptosis.	Less expression/activity → more sensitivity	[29]
	Hematological malignancies (patient biopsies, primary cells and cell lines)	Increased expression in acute myeloid leukemia patients predictive of a poor outcome; Kv10.1 inhibition by blockers or siRNA reduced cell proliferation and increased sensitivity to etoposide, cytarabine, or doxorubicin by promoting caspase activity.	Less expression/activity → more sensitivity	[30]
Kv1.5	Gastric cancer (cell lines)	Kv1.5 inhibition by K^+^ channel blocker or siRNA enhanced resistance to doxurubicin, 5-fluouracil, vincristine, or cisplatin, while Kv1.5 overexpression increased chemosensitivity.	Less expression/activity → less sensitivity More expression/activity → more sensitivity	[31]
Kv1.1, Kv1.3	Cancer cell line panel	Expression positively correlated with cisplatin-induced cell death.	More expression/activity → more sensitivity	[32]
KCa1.1 (BK)	Ovarian cancer (cell lines and primary cells)	KCa1.1 expression is inversely correlated with resistance to cisplatin; Channel knockdown by siRNA increased resistance to cisplatin.	Less expression/activity → less sensitivity	[33]
	Glioblastoma (cell line)	KCa1.1 promotes hypoxia-induced cell migration and resistance to cisplatin; KCa1.1 inhibition by paxilline increased sensitivity to cisplatin.	More expression/activity → less sensitivity Less expression/activity → more sensitivity	[34]
Kir2.1	Small cell lung cancer (patients, cell lines, and in vivo mouse model)	Increased Kir2.1 expression in patients’ cancer cells correlated with clinical stage progression and chemoresistance; Overexpression of Kir2.1 increased resistance to etoposide or cisplatin, whereas knockdown with siRNA increased chemosensitivity.	More expression/activity → less sensitivity Less expression/activity → more sensitivity	[35]
